# Minieditorial: A Hidroterapia Reduz a Rigidez Arterial em Gestantes Hipertensas Crônicas

**DOI:** 10.36660/abc.20200251

**Published:** 2020-05-12

**Authors:** Celso Amodeo

**Affiliations:** Universidade Federal de São Paulo São Paulo SP Brasil Universidade Federal de São Paulo (UNIFESP), São Paulo, SP – Brasil

**Keywords:** Gestantes/complicações, Hipertensão, Pré-Eclampsia, Velocidade de Onda de Pulso, Rigidez Arterial, Pressão Arterial, Hidroterapia/métodos

Com o envelhecimento, observa-se um aumento na pressão de pulso principalmente às custas do aumento da pressão arterial sistólica, mas também em parte pela redução da pressão diastólica. Essa, quando abaixo de 50 mmHg, demonstra relação com o aumento do risco cardiovascular.

Com o desenvolvimento de aparelhos que inferem de forma indireta a pressão central na raiz da aorta e oferecem a possibilidade de análise da rigidez arterial (outro marcador de risco cardiovascular), muito trabalhos têm demonstrado maior precocidade de alterações na dinâmica vascular com as medidas centrais em comparação com medidas periféricas da pressão arterial.

A velocidade de onda de pulso permite analisar a rigidez arterial e a obtenção de um parâmetro chamado índice de incrementação (tradução do inglês augmentation index) corrigido pela frequência cardíaca de 75 bpm (AIx@75) que também se correlaciona com maior rigidez arterial e risco cardiovascular.

O estudo de Linhares et al.,^1^ trata da análise das medidas de pressão central em mulheres grávidas com ou sem hipertensão arterial que são submetidas a sessões de hidroterapia. Trata-se de um estudo interessante visto que ainda hoje se discute muito a fisiopatologia da pré-eclâmpsia e eclampsia. Alguns autores têm observado comportamentos diferentes desses parâmetros centrais entre as pacientes que apresentam uma gravidez normal e aquelas que desenvolvem eclampsia ou pré-eclâmpsia.

Na discussão deste artigo está citado o trabalho de Yinon et al.,^2^ que observaram um AIx@75 elevado seis a 24 meses após o parto em mulheres que tiveram pré-eclâmpsia ou conceptos de baixo peso. Tomimatsu et al.^3^ também observaram que o AIx@75 durante 26 a 32 semanas de gestação teve maior correlação com o peso ao nascimento do que a pressão arterial medida no braço, inferindo que o AIx@75 seria um parâmetro hemodinâmico importante que se identifica em grávidas que desenvolvem restrição do crescimento fetal.

Partindo dessas informações, os autores estudaram o efeito da hidroterapia em mulheres grávidas sobre os parâmetros de medida central, pressão arterial central sistólica e diastólica, AIx@75 e velocidade de onda de pulso. Com esses parâmetros foi possível observar o comportamento da rigidez arterial e sua resposta aos exercícios de hidroterapia. Ficou demonstrado que agudamente a hidroterapia reduz o AIx@75 tanto em grávidas hipertensas quanto em normotensas. Todavia, não houve alteração da velocidade da onda de pulso e das pressões arteriais medidas no braço. Há que se considerar a análise de quanto tempo esse efeito de redução do AIx@75 persiste e se realmente isso teria um impacto na prevenção das complicações hipertensivas da gravidez, mas isso não foi analisado nesse trabalho. Entretanto, não deixa de abrir um campo para futuras investigações de um potencial mecanismo fisiopatológico da pré-eclâmpsia e da eclampsia. Além disso, os resultados apontam para o potencial uso da hidroterapia como um instrumento de identificação mais precoce de mulheres grávidas em risco para o desenvolvimento de complicações produzidas pela hipertensão arterial.
