# Supplementary material for: Minieditorial: A Hidroterapia Reduz a Rigidez Arterial em Gestantes Hipertensas Crônicas
Source: Arq Bras Cardiol. 2020 May 12;114(4):655. [Article in Portuguese] doi: 10.36660/abc.20200251 (PMC9744349; doi:10.36660/abc.20200251)
Supplement: Translation

# Translation: Short Editorial: Hydrotherapy Reduces Arterial Stiffness in Pregnant Women with Chronic Hypertension

**Authors:** Celso Amodeo

**Affiliations:** Brazil Universidade Federal de São Paulo (UNIFESP), São Paulo, SP – Brazil

**Keywords:** Pregnant Women/complicações, Hypertension, Pre-Eclampsia, Pulse Wave Analysis, Blood Pressure, Vascular Stiffness, Hydrotherapy/methods

Increased pulse pressure is associated with aging. This is mainly caused by increased systolic pressure, but also by decreased diastolic pressure. Diastolic blood pressure below 50 mmHg has been related to increased cardiovascular risk.

With the development of devices that allow the indirect estimation of central pressure from the aortic root and analysis of arterial stiffness (another marker of cardiovascular risk), many studies have shown that changes in vascular dynamics are detected earlier using central measurements compared with peripheral blood pressure measurements.

Assessment of pulse wave velocity allows the analysis of arterial stiffness and determination of the augmentation index, which corrected by the heart rate of 75 bpm (AIx@75), is associated with greater arterial stiffness and higher cardiovascular risk.

The study by Linhares et al.^1^ reports the results of central pressure measurements in pregnant women with and without hypertension undergoing hydrotherapy. This is an interesting study considering that the pathophysiology of preeclampsia and eclampsia is still on debate. Some authors have reported different behaviors of these central blood pressure parameters in normal pregnancy and in women with eclampsia/preeclampsia.

In the discussion section, the authors cited the study by Yinon et al.^2^ who observed an increased AIx@75 in women with previous early-onset preeclampsia and intrauterine growth restriction. Tomimatsu et al.^3^ also reported that an AIx@75 measured during 26–32 weeks of gestation showed a stronger correlation with birth weight than brachial blood pressure, indicating that the AIx@75 is a relevant hemodynamic parameter in pregnant women with intrauterine growth restriction.

From this information, Linhares et al. investigated the effects of hydrotherapy on central blood pressure parameters, central systolic and diastolic blood pressure, AIx@75 and pulse wave velocity in pregnant women. The authors observed the behavior of arterial stiffness in response to hydrotherapy. The acute effect of the therapy was a reduction in AIx@75 in both hypertensive and normotensive pregnant women, but no changes were seen in pulse wave velocity or brachial blood pressure. The authors did not evaluate, however, the duration of AIx@75 reduction, and whether this effect would have an impact on preventing hypertensive complications in pregnancy. This opens the possibility for future studies on the pathophysiological mechanisms of preeclampsia and eclampsia. Also, the results indicate the use of hydrotherapy as an instrument for early detection of pregnant women at risk for development of hypertensive complications.
